# Coronary artery calcifications predict long term cardiovascular events in non diabetic Caucasian hemodialysis patients

**DOI:** 10.18632/aging.100740

**Published:** 2015-04-24

**Authors:** Annalisa Noce, Maria Paola Canale, Ambrogio Capria, Valentina Rovella, Manfredi Tesauro, Giorgio Splendiani, Margherita Annicchiarico-Petruzzelli, Micol Manzuoli, Giovanni Simonetti, Nicola Di Daniele

**Affiliations:** ^1^ Hypertension and Nephrology Unit, Department of Systems Medicine, University of Rome ”Tor Vergata”, Rome Italy; ^2^ Lazio Regional Agency for Transplantations and Related Pathologies, Rome Italy; ^3^ Biochemistry Laboratory, IDI-IRCCS, c/o Department of Experimental Medicine and Surgery, University of Rome “Tor Vergata” Italy; ^4^ Department of Diagnostic Imaging, Molecular imaging, Interventional Radiology and Radiotherapy, University of Rome ”Tor Vergata”, Rome Italy

**Keywords:** aging, cardiac calcifications, spiral computed tomography, mortality, morbidity, cardiovascular events, vascular pathology, endothelial cells, hemodialysis

## Abstract

Vascular calcifications are frequent in chronic renal disease and are associated to significant cardiovascular morbidity and mortality. The long term predictive value of coronary artery calcifications detected by multi-layer spiral computed tomography for major cardiovascular events was evaluated in non-diabetic Caucasian patients on maintenance hemodialysis free of clinical cardiovascular disease. Two-hundred and five patients on maintenance hemodialysis were enrolled into this observational, prospective cohort study. Patients underwent a single cardiac multi-layer spiral computed tomography. Calcium load was quantified and patients grouped according to the Agatston score: group 1 (Agatston score: 0), group 2 (Agatston score 1-400), group 3 (Agatston score 401-1000) and group 4 (Agatston score >1000). Follow-up was longer than seven years. Primary endpoint was death from a major cardiovascular event. Actuarial survival was calculated separately in the four groups with Kaplan-Meier method. Patients who died from causes other than cardiovascular disease and transplanted patients were censored. The “log rank” test was employed to compare survival curves. One-hundred two patients (49.7%) died for a major cardiovascular event during the follow-up period. Seven-year actuarial survival was more than 90% for groups 1 and 2, but failed to about 50% for group 3 and to <10% for group 4. Hence, Agatston score >400 predicts a significantly higher cardiovascular mortality compared with Agatston score <400 (p<0.0001); furthermore, serum Parathyroid hormone levels > 300 pg/l were associated to a lower survival (p < 0.05). Extended coronary artery calcifications detected by cardiac multi-layer spiral computed tomography, strongly predicted long term cardiovascular mortality in non-diabetic Caucasian patients on maintenance hemodialysis. Moreover, it was not related to conventional indices of atherosclerosis, but to other non-traditional risk factors, as serum Parathyroid hormone levels. A full cost-benefit analysis is however necessary to justify a widespread use of cardiac multi-layer spiral computed tomography in clinical practice.

## INTRODUCTION

Dialysis patients exhibit an increased all-cause and cardiovascular (CV) mortality when compared to the general aging population [1]. In particular, with the increased aging of the population, calcification of vessels and cardiac valves is highly prevalent in maintenance hemodialysis (mHD) patients and has been associated with an increased CV risk as well as with all-cause mortality [2]. In patients with end stage renal disease (ESRD), besides traditional risk factors such as increased age, hypertension (HT), diabetes (DM) and dyslipidemia, non-traditional CV risk factors, such as mineral metabolism abnormalities, extreme PTH serum levels, excessive administration of calcium salts as phosphate binders, chronic inflammation, malnutrition and oxidative stress play an important role in development of CV diseases [3].

In previous studies, male gender, dialysis vintage, smoking, calcium-phosphate product, high serum high-sensitivity C reactive protein, low Kt/V_urea_, Diabetes Mellitus and ethnicity were independent risk factors for CV calcifications [4,5]. Type 2 diabetic patients show an increased risk of CV events that is similar to the risk of non-diabetic patients with coronary artery disease [6], regardless of other concomitant CV risk factors [7]. Moreover, both type 1 and type 2 DM are frequently complicated by renal disease [8] and kidney disease in diabetic patients dramatically increases the incidence of CV events [9-12]. Therefore, renal disease must be considered an independent risk factor for CV disease at least as strong as DM. Indeed, regardless of DM, gender and ethnicity, incident HD patients show a CV morbidity/mortality increased by ten to one hundred times compared to the age-matched general population: CV disease-related mortality rate of a 25 to 35 year-old remic patient may be similar to that of people 85 or more years old [13].

The primary aim of this study was to evaluate the long term predictive value for CV events of coronary artery calcifications (CACs) detected by multi-layer spiral computed tomography (MSCT) in non-diabetic Caucasian mHD patients.

The secondary aim of the study was then to evaluate whether conventional “atherogenic indices” were an independent risk factor for CACs. The following “atherogenic indices” were investigated: total cholesterol (TC)/high-density lipoprotein (HDL) cholesterol, low-density lipoprotein (LDL) cholesterol/HDL cholesterol and Triglyceride Logarithm/HDL cholesterol.

## RESULTS

Demographic, clinical features and laboratory findings of the study population divided on AS values <400 vs. >400 are summarized in Table 1. We selected 400 AS units as cut-off value according to previous literature [14].

**Table 1:** Demographic, clinical and laboratory characteristics of study population by agatstone score

	AS ≤ 400	AS >400	p (Fisher Test)
***Demographics***			
**Patients**	72/205 (35,1%)***	133/205 (64,9%)***	
**Age (years)**	52,50±12,65*	63,98±10,82*	0,126
**Female**	34/72	41/133	0,119
**Male**	38/72	92/133	0,236
***Clinical Characteristics***			
**BM**	23,98±4,80*	24,81±3,77*	0,039
**Hypertension**	22/72 (30,5%)***	55/133 (41,4%)***	
**HD vintage (months)**	37 (6-276)**	48 (6-252)**	0,579
**Kt/Vurea**	1,31 (1-1,75)**	1,28 (0,85-1,84)**	0,957
***Laboratory data***			
**CPR (mg/l)**	1,12±1,09	1,08±1,19	0,885
**Hemoglobin (g/dl)**	11,47±1,09*	11,61±1,2*	0,472
**Calcium (mmol/l)**	9,43±0,77*	9,47±1,01*	0,120
**Phosphorus (mmol/l)**	6,00±1,19*	5,62±1,34*	0,258
**Ca × P**	55,41±12,50*	53,19±14,52*	0,166
**iPTH (pg/l)**	383 (61-1498)**	451 (2,43-2500)**	0,015
**Total cholesterol (mmol/l)**	164,83±45,95*	157,12±38,42*	0,080
**HDL cholesterol (mmol/l)**	48,2±14,53*	42,90±12,70*	0,188
**LDL cholesterol (mmol/l)**	79,50 (38-311,4)**	93 (40-254,2)**	0,032
**Triglycerides (mmol/l)**	143 (50-747)**	171 (53-716)**	0,796
**Serum Albumin (g/l)**	4,23±0,30*	4,12±0,39*	0,043

**Abbreviations**: AS, Agatstone Score; HD, hemodialysis; Ca, calcium; P, phosphorus; iPTH, Intact Parathyroid Hormone; LDL, Low-Density Lipoprotein; HDL, High-Density Lipoprotein.

**Note**

* Normally distributed data are expressed as mean ± standard deviation

** Non normally distributes data are expressed as median with ranges

*** Data are expressed as percentage

Among the 205 mHD patients enrolled into the study, 72 (35.1%) showed an AS ≤ 400 units and 133 (64.9%) an AS >400 units. Age, HD-vintage, Kt/V_urea_, C-reactive protein (CRP), serum haemoglobin (Hb), glucose, calcium, phosphorus and calcium-phosphorus product, fibrinogen, total cholesterol, HDL cholesterol and serum triglycerides did not differ significantly in patients with AS above or below 400. Statistically significantly higher values were found in patients with AS >400 units for BMI (p=0.039) (values less than 25 in both groups), serum intact parathyroid hormone (iPTH) (p=0.015) and serum LDL cholesterol (p=0.032) (values less than 100mg/dl in both groups), whereas serum albumin was statistically lower (p=0.043) in patients with AS >400 units but still above 4mg/dl.

Drug therapy for dyslipidemia and for the management of calcium/phosphorus metabolism of study population did not differ in patients with AS above or below 400 (NS) (Table 2).

**Table 2:** Drug therapy of patients

	AS ≤ 400 (n=72)	AS >400 (n=133)	p value
**1.Phosphate binders**:			
**a-calcium carbonate**	19,44% (n=14)	20,30% (n=27)	0,495
**b-sevelamer**	65,28% (n=47)	67,67% (n=90)	0,225
**2.Vitamin D analogues**:	80,55% (n=58)	81,95% (n=109)	0,531
**3.Statins**:	58,33% (n=42)	56,31% (n=75)	0,589

The “atherogenic indices” or lipoprotein ratio stratified by AS values, in male and female patients, are described in Tables 3 A and B, respectively. In male patients, no statistically significant differences were observed for TC/HDL-cholesterol ratio, LDL/HDL cholesterol ratio and Logarithmic transformation of the triglyceride/HDL cholesterol molar concentration ratio in patients with AS above or below 400. Moreover, all parameters were in the normal range. In female patients, TC/HDL-cholesterol ratio did not differ in patients with AS above or below 400. Although a statistical difference was observed for both LDL/HDL cholesterol ratio and Logarithmic transformation of the triglyceride/HDL cholesterol molar concentration ratio in patients with AS above or below 400, these values were in the normal range.

**Table 3A:** Atherogenic indices values by Agatstone score in male

	AS≤400	AS>400	p value
**TC/HDL-C**	3,73±1,28^a^	3,90±1,40^a^	0,52*
**LDL-C/HDL-C**	2,47±1,78^a^	2,85±1,6^a^	0,0663
**Log Trigl/HDL col**	0,07±0,10^a^	0,07±0,09^a^	0,9453*

**Table 3B:** Atherogenic indices values by Agatstone score in female

	AS≤400	AS>400	p value
**TC/HDL-C**	3,29±1,09^a^	3,79±1,33^a^	0,09
**LDL-C/HDL-C**	1,83±0,79^a^	2,52±1,27^a^	0,0074
**Log Trigl/HDL col**	0,04±0,01^a^	0,05±0,16^a^	0,009*

a Data are expressed as mean ± standard deviation.

P<0,05 is considered statistical significant.

* We applied Mann/Whitney Test

**Abbreviation**: HDL-C, High-Density Lipoprotein Cholesterol; LDL-C, Low-Density Lipoprotein Cholesterol; TC, Total Cholesterol; Log Trigl, Triglyceride Logarithm.

No correlation was found between serum iPTH levels and AS value (r =-0.046, p=0.64).

### Survival analysis

**Figure 1:**
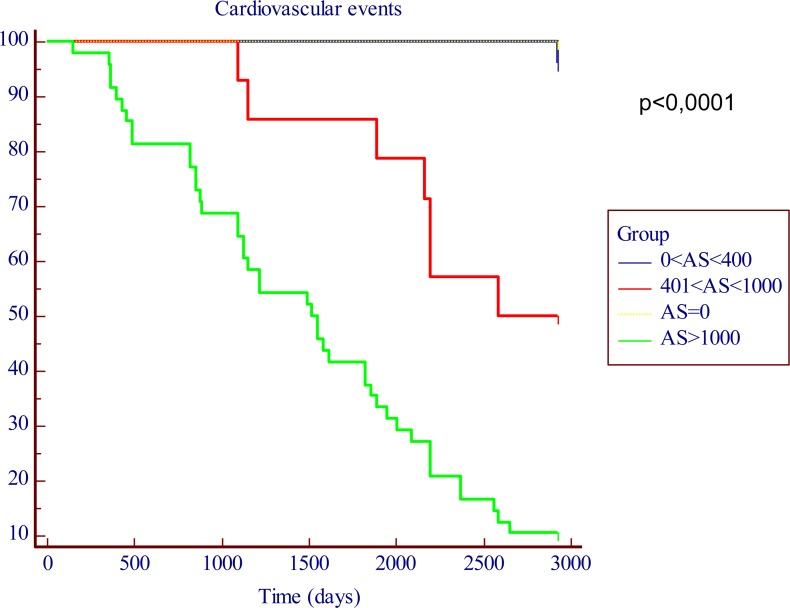
Kaplan-Meier in function of the AS cut-off levels. The time is expressed as days.

One-hundred two patients (49.7%) had a CV event during the seven year follow-up period. Kaplan-Meier survival analysis is shown in Figure 1. Seven-year actuarial survival exceeded 90% among patients of groups 1 (AS=0) and 2 (AS≤400), but fell to about 50% in patients of group 3 (400<AS≤1000) and to less than 10% in patients of group 4 (AS>1000). Hence, patients with AS>400 showed a significantly higher CV morbidity/mortality compared to patients with AS≤400 (p<0.0001).

Seven-year actuarial survival exceeded 90% among patients of groups 1 (AS=0) and 2 (AS≤400), but fell to about 50% in patients of group 3 (400<AS≤1000) and to less than 10% in patients of group 4 (AS>1000). Hence, patients with AS>400 showed a significantly higher CV morbidity/mortality compared to patients with AS≤400 (p<0.0001) (Figure 1).

**Figure 2:**
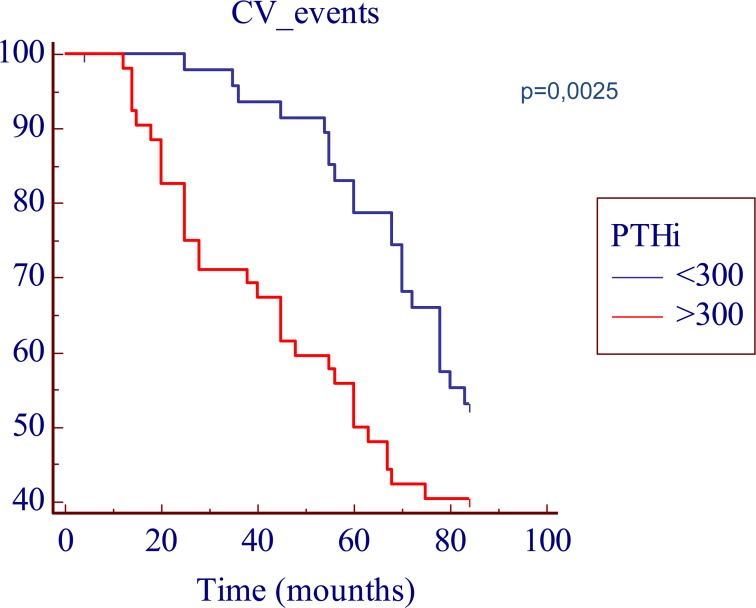
Kaplan-Meier survival plot in function of the iPTH cut-off levels. The time is expressed as months.

We found a predictive role, both for an higher AS and an higher CV mortality, linked to the serum iPTH levels; in fact, as shown in Table 1, the iPTH values were significantly higher in pts with an AS > 400 (451 pg/L, [range 2.43 to 2500 pg/L] vs 383 pg/L [range 61 to 1498 pg/L], p < 0.05); furthermore the Kaplan-Meier analysis showed a significantly decreased seven-year actuarial survival (p < 0.05) in patients with iPTH levels > 300 pg/L compared to patients with iPTH levels < 300 pg/L (Figure 2).

Nine out of the 205 patients enrolled into the study were censored: four died for non-CV causes and 5 underwent renal transplantation.

## DISCUSSION

With the changing medical treatments and with the changes in the living environment, the age of the population is increasing in all countries, posing novel challenges to the medical and social areas [15-27]. While the changes related to increased age implies significant problems for the society, relevant modifications needs to be clarified for the physical, biological and mental health. This has fostered important research in distinct medical aspects, from the major role played by the telomers [28,29] to the study in different animal models, from bacteria, C. elegans to drosophila or mice where the cell autonomy and the underlying molecular pathways have been deeply investigated [30-39]. Recently it has become apparent that the complex genetic and epigenetic gene regulation in aging is also affected by a large array of microRNA [40-48], that contribute to the cellular signalling [49-58], which in turn regulate several cellular function, such as for example metabolism [59-64], cell division [65-66], or DNA damage [67-70]. A significant expansion of the latter is the involvement of p53 [71-76] and its family members p63 [77-79] and p73 [80-83] with their metabolic regulation. All these biochemical alterations, profoundly affect different organs, such as for example the neural [84-95] and the cardiovascular [96,97] systems. Accordingly, in the present manuscript we investigated a specific aspect of vascular biology.

Calcification of vessels and cardiac valves is highly prevalent in maintenance hemodialysis (mHD) patients and has been associated with an increased CV risk as well as with all-cause mortality [2].

Vascular calcifications can affect the media or intima. Medial artery calcifications are frequently found in DM and uremia [98]. Vascular calcifications are associated with adverse clinical outcomes [99]. Hemodynamic disturbances related to vascular calcifications include loss of arterial elasticity, an increased arterial stiffness and pulse wave velocity, with subsequent development of myocardial ischemia and heart failure [100].

Pathogenesis of vascular calcifications is complex. It does not only consist in a simple precipitation of calcium and phosphate, but is instead an active process in which vascular smooth muscle cells (VSMCs), undergo apoptosis and vesicles formation, are transformed into osteoblast-like cells, inducing both matrix formation and attracting local factors involved in the mineralization process [100].

Jono et al. suggested that elevated intracellular phosphate may directly stimulate VSMCs to transform into calcifying cells by activating genes associated with osteoblastic function [101]. Hyperfosfatemia is responsible for the enhanced number and activity of osteoclasts, being a major contributor to increased bone resorption in chronic kidney disease [102].

Cardiac calcifications predict CV events and all-cause mortality in non diabetic and add incremental predictive value to conventional CV risk factors [103].

In the present prospective, cohort and long-term study we assessed CACS by using a semi-quantitative calcification score. In patients with CACS less than 400 AS units seven-year actuarial survival was very high (more than 90%) compared to patients with AS> 400. A striking increase in long term CV morbidity/mortality associated with the increase of baseline CACS was observed. Seven-year actuarial survival dropped to less than 10% in patients with extensive calcifications (AS>1000).

We found a significant predictive role, both for an higher AS and for the serum iPTH levels; in fact, the seven year actuarial survival was lower both in patients with an AS > 400 and in patients with iPTH levels > 300.

Other studies have addressed this issue using less invasive methodology to assess CV calcifications in both diabetic and non-diabetic mHD population [104]; in contrast with the results of previous studies, age, male gender, dialysis vintage, calcium-phosphate product, CRP serum levels, serum albumin, Kt/V_urea_, were not independent risk factors for CACS in our non-diabetic study population, suggesting that the more accurate, semi-quantitative method used for measurement, the anatomic location and the exclusion of diabetic patients probably avoided confounding factors. Interestingly, “atherogenic indices” were not independent risk factors for CACS and LDL cholesterol was at target levels regardless to the extent of cardiac calcifications suggesting other factors to be important for CACS formation.

Our findings corroborate the well-known poor impact of traditional cardiovascular risk factor in non diabetic hemodialysis patients, and support that usual risk factors, as well as age, blood pressure and lipid levels are working with other factors, in particular iPTH levels, to contribute to the development and progression of vascular calcifications. It is worthy to note that higher serum iPTH levels are strongly associated with the presence of cardiac calcification, because a deregulated calcium-phosphorus is as important long term CV risk factor in non diabetic mHD patients and stress the importance of optimal calcium-phosphorus metabolism control in order to prevent or reduce the occurrence of CV events [105].

Although accurate MSCT is more invasive and the ionizing radiation dose is higher compared to roentgenography, it is likely that patients with higher iPTH and phosphorus levels could be suitable for screening and widespread clinical use in asymptomatic patients, may although provide additional information in situations in which a more accurate assessment of CV risk would be necessary.

## CONCLUSIONS

Following our previous interest on aging [106-108], here we show that extensive coronary artery calcifications strongly predict long term CV morbidity and mortality in non diabetic Caucasian mHD patients independently of atherogenic indices. A full cost benefit analysis would be needed before more widespread screening of MSCT could be advocated in clinical practice, employing a CV risk stratification plan that includes iPTH with other usual stronger atherogenic indices, linked to cholesterol and triglycerides ratios.

## MATERIALS AND METHODS

### Study design and patients

This prospective and cohort study was designed specifically to evaluate CACS as a possible predictor of CV events in mHD patients with no evidence of cardiovascular disease. Patients recruited from five dialysis facilities, had coronary artery calcifications measured by multi-layer spiral computed tomography (MSCT). All of them were performed at our university centre. Clinical data, features of dialytic treatment and information on outcome were provided for each patient at regular six-month interval. Recruitment started in January 2003. Participants' clinical status was annually reviewed annually up to January 2011.

The study protocol complied with the declaration of Helsinki and was appointed by our local ethical committee and by ethical committee of each of the participating institutions. A written fully informed consent was provided by all patients before entering the study.

Men and women aged 18 years or more, on mHD since 6 months at least were considered eligible for the study.

Exclusion criteria were: diabetes, pregnancy, known coronary artery disease, congestive heart failure, uncontrolled HT, cerebro-vascular ischemic events in the last six months, cardiac arrhythmias (that would impede assessment of CACS with computer tomography), neoplastic disease, inability to provide informed consent or other medical conditions likely to limit life expectancy or requiring extensive medical treatment.

Two-hundred five (105 male and 100 female) Caucasian mHD patients were enrolled into the study. At enrolment time each patients performed the coronary MSCT. Mean age was 59.85±12.77 years, HD vintage was 62.30±55 months.

All patients received standard bicarbonate dialysis with1.5–2.0m^2^ hollow-fiber low-flux polysulphone membranes *(Lo-PS Diacap Polysulphone, B. Braun gmbh, Melsungen, Germany*), four hours three times weekly, through a well-functioning native A-V fistula or a cuffed internal jugular indwelling venous catheter. The vascular access performance was satisfactory with a blood flow of at least 300ml/min and a Kt/V _urea_ ratio>1.3 [109].

Body mass index (BMI) was calculated upon the post-dialysis body weight (“dry weight”) at enrollment by dividing weight in kilograms by the square of height in meters for every patient.

### Laboratory measurements

After an overnight fast blood samples were taken for baseline measurements of serum glucose, serum Hb, total and HDL cholesterol, (LDL cholesterol was calculated by Friedewald method), triglycerides, serum Calcium and Phosphorus, iPTH, fibrinogen and CRP. Blood samples were drawn in a midweek non-dialysis day.

Atherogenic indices were calculated as follows: total cholesterol (mg/dl) /HDL cholesterol (mg/dl) (normal value <5 for male and <4.5 for female), LDL cholesterol (mg/dl)/HDL cholesterol (mg/dl) (normal value<3.5 for male and <3 for female). An atherogenic plasma index [log (triglycerides mg/dl/HDL cholesterol mg/dl)], >0.5 has been proposed as cut-off point indicating atherogenic risk. Risk categories and target levels for total cholesterol/HDL-cholesterol, LDL-cholesterol/HDL-cholesterol in primary and secondary prevention, stratified by gender, were considered [110].

### Cardiac calcifications

Quantification of CACS was obtained with a coronary MSCT. The measurement of CACS was expressed in Agatston score (AS) units [111].

MSCT was performed with a 64-channel multidetector scanner (LightSpeed VCT, General Electric Medical Systems, EU) and retrospective synchronization technique. Images were acquired without enhancement with the patient in the supine position. All scans were performed with the following parameters: detector collimation 4 × 2.5mm, reconstruction interval 10mm, gantry rotation time 0.5 sec, tube voltage 120Kv, intensity 300mA, field of view 25cm, cranio-caudal scan direction.

Calcium was scored according to the Agatston method to quantify the amount of calcification in the coronary arteries.

Coronary calcification was defined as the presence of four or more contiguous pixels with more than 130 Hounsfield units (HU). The investigator scored each of the 20 slices individually, using GE Medical Systems-Advantage Workstation software. Thereby, each plaque score was generated as the product of the area and density. This method has previously been described in detail by Agatston et al. [112,113]. According to the AS patients were then divided in four groups: group 1(AS=0), group 2 (0<AS≤400), group 3 (400<AS≤1000) and group 4 (AS>1000).

### Clinical endpoints

Primary clinical endpoint was a fatal major CV event: death due to myocardial infarction (MI), heart failure or other CV causes (aortic aneurysm rupture, stroke, i.e.) during the 7 year follow-up period following baseline evaluation. Clinical outcome was assessed by direct interview contact to the patients and inspection of medical or other records.

### Statistical analysis

Results related to biochemical and radiological findings are expressed as the mean ± standard deviation (SD) of three independent determinations. Two-tailed independent-sample T test and two sided Fisher's exact test were employed for analysis of results; p-values <0.05 were considered statistically significant.

Seven-year actuarial survival was calculated in the four groups separately by Kaplan-Meier method. The “log rank” test was employed to compare survival curves. Patients who died for causes other than CV diseases and transplanted patients were censored. To study the linear relationship between i-PTH levels and AS values, non parametric correlation (Spearman p) was used. Data were elaborated through the MedCalc Statistical Software (MedCalc Software, 9030 Mariakerke, Belgium).
